# Resveratrol Increases Nitric Oxide Production in the Rat Thick Ascending Limb via Ca^2+^/Calmodulin

**DOI:** 10.1371/journal.pone.0110487

**Published:** 2014-10-14

**Authors:** Agustin Gonzalez-Vicente, Pablo D. Cabral, Jeffrey L. Garvin

**Affiliations:** 1 Department of Physiology and Biophysics, Case Western Reserve University, School of Medicine, Cleveland, Ohio, United States of America; 2 Universidad de Buenos Aires, Facultad de Farmacia y Bioquímica, Ciudad Autónoma de Buenos Aires, Buenos Aires, Argentina; 3 Universidad de Buenos Aires, Facultad de Medicina, Ciudad Autónoma de Buenos Aires, Buenos Aires, Argentina; 4 Hypertension and Vascular Research, Department of Internal Medicine, Henry Ford Hospital, Detroit, Michigan, United States of America; Emory University, United States of America

## Abstract

The thick ascending limb of the loop of Henle reabsorbs 30% of the NaCl filtered through the glomerulus. Nitric oxide (NO) produced by NO synthase 3 (NOS3) inhibits NaCl absorption by this segment. Resveratrol, a polyphenol, has beneficial cardiovascular and renal effects, many of which are mediated by NO. Resveratrol increases intracellular Ca^2+^ (Ca_i_) and AMP kinase (AMPK) and NAD-dependent deacetylase sirtuin1 (SIRT1) activities, all of which could activate NO production. We hypothesized that resveratrol stimulates NO production by thick ascending limbs *via* a Ca^2+^/calmodulin-dependent mechanism. To test this, the effect of resveratrol on NO bioavailability was measured in thick ascending limb suspensions. Ca_i_ was measured in single perfused thick ascending limbs. SIRT1 activity and expression were measured in thick ascending limb lysates. Resveratrol (100 µM) increased NO bioavailability in thick ascending limb suspensions by 1.3±0.2 AFU/mg/min (*p*<0.03). The NOS inhibitor L-NAME blunted resveratrol-stimulated NO bioavailability by 96±11% (*p<*0.03). The superoxide scavenger tempol had no effect. Resveratrol elevated Ca_i_ from 48±7 to 135±24 nM (*p*<0.01) in single tubules. In Ca^2+^-free media, the resveratrol-induced increase in NO was blunted by 60±20% (*p*<0.05) and the rise in Ca_i_ reduced by 80%. Calmodulin inhibition prevented the resveratrol-induced increase in NO (*p<*0.002). AMPK inhibition had no effect. Resveratrol did not increase SIRT1 activity. We conclude that resveratrol increases NO production in thick ascending limbs *via* a Ca^2+^/calmodulin dependent mechanism, and SIRT1 and AMPK do not participate. Resveratrol-stimulated NO production in thick ascending limbs may account for part of its beneficial effects.

## Introduction

Defective NO signaling in thick ascending limbs contributes to several forms of salt-sensitive hypertension [1]–[3]. Thick ascending limbs express all three nitric oxide synthase isoforms (NOS1, 2 and 3). However, only NO produced by NOS3 has been shown to inhibit NaCl reabsorption in this segment [4], [5] contributing to diuresis and natriuresis [6].

Originally NOS3 was reported to be activated by increases in intracellular Ca^2+^ (Ca_i_) which cause the Ca^2+^/calmodulin complex to associate with the enzyme. Binding of Ca^2+^/calmodulin to NOS3 facilitates the electron flux through its domains and increases NO production [7], [8]. However, several stimuli such as luminal flow, ATP, endothelin and angiotensin II stimulate NOS3 by enhancing phosphatidylinositol 3 kinase and Akt activity. Akt phosphorylates of NOS3 at the serine 1177 (S1177) which reduces dissociation of calmodulin at low Ca_i_ levels, such that normal basal Ca_i_ is sufficient to increase NO production. Other kinases such as AMP kinase (AMPK) also phosphorylate NOS3 at S1177, activating it independently of increases in Ca_i_ [8]. In addition, deacetylation of the lysines 496 and 506 within the calmodulin binding domain by NAD-dependent deacetylase sirtuin1 (SIRT1) is thought to favor calmodulin binding and NOS3 activation [9].

The polyphenol resveratrol is abundant in the skin of *Vitis vinifera* grapes, and remains present at high concentrations in red wines [10]. This molecule is thought to be responsible for the beneficial effects of red wine consumption. Resveratrol is rapidly cleared from the blood stream mainly by the kidney where it remains elevated for several hours [11]–[14]. Resveratrol has beneficial cardiovascular effects in humans [15]–[17] including reductions in blood pressure [18], [19]. It improves renal functional and histological parameters in several animal models of kidney damage by oxidative stress [20]–[24]. However, unlike other phenolic compounds such as gallic or caffeic acids, the free radical scavenging capacity of resveratrol is low [25], [26], as is its contribution to the overall antioxidant capacity of wine [27]. Thus its beneficial actions in the kidney are thought to be mediated by the L-arginine/NO/cGMP pathway [20], [21].

Although NO mediates many of the effects of resveratrol, the mechanisms by which it activates NOS3 and increases NO are not fully understood, and may be tissue dependent. Resveratrol has been reported to stimulate AMPK [23], [28] and SIRT1 [29], [30] activities, both of which can activate NOS3. It has also been reported to increase NO production by raising Ca_i_ [31]. Resveratrol alters Akt activity [24], [32], but this is an inhibitory effect which would tend to decrease rather than increase NOS3 activity. Finally, even though unlikely due to its poor antioxidant capacity, resveratrol could increase NO bioavailability by reacting with reactive oxygen species [25]–[27] that scavenge NO. We hypothesize that resveratrol stimulates NO production by thick ascending limbs *via* a Ca^2+^/Calmodulin-dependent mechanism.

## Materials and Methods

### Animals

This study was approved by the Case Western Reserve University and the Henry Ford Hospital Institutional Animal Care and Use Committees. All experiments were conducted in accordance with the National Institutes of Health Guidelines for the Care and Use of Laboratory Animals. Male Sprague-Dawley rats (Charles River Breeding Laboratories, Wilmington, MA) weighing between 220–260 g were anesthetized with ketamine (100 mg/kg bw IP) and xylazine (20 mg/kg bw IP), and given 2 IU heparin (IP). All efforts were made to minimize suffering. Animals were sacrificed while still under anesthesia.

### Drugs and buffers

Unless specified, all drugs and reagents were obtained from Sigma-Aldrich [St Louis, MO]. The cell-permeable NO-selective fluorescent dye DAF-FM-diacetate, the Ca^2+^-sensitive dye FURA 2-AM, and the Ca^2+^ ionophore 4-Bromo A-23187 (4Br-A23187) were obtained from Invitrogen [Grand Island, NY]. Coomasie Plus Protein Assay Reagent was obtained from Thermo-Scientific, [Rockford, IL].

HEPES-buffered physiological saline (2 mM Ca^2+^ media) contained [in mmol/l]: 10 HEPES (4-(2-hydroxyethyl)-1-piperazineethanesulfonic acid) (pH 7.5), 130 NaCl, 4 KCl, 2.5 NaH_2_PO_4_, 1.2 MgSO_4_, 5.5 glucose, 6.0 DL-alanine, 2.0 Ca(lactate)_2_, and 1.0 Na_3_citrate. Ca^2+^-free media contained [in mmol/l]: 10 HEPES (pH 7.5), 130 NaCl, 4 KCl, 1.2 MgSO_4_, and either 100 or 200 µmol/l ethylene glycol tetraacetic acid (EGTA). Osmolalities of both solutions were adjusted to 300±5 mOsmol/l with mannitol.

### Thick ascending limb suspensions

Suspensions were prepared as follows: kidneys were perfused retrograde *via* the abdominal aorta with cold HEPES-buffered physiological saline containing 2.5 U/ml heparin and 0.1% Type I collagenase. Perfused kidneys were removed, coronal slices cut and outer medullary tissue dissected and minced. Minced tissue was digested in 0.1% collagenase for 30 min at 37°C. During digestion, tissue was agitated and gassed with 100% O_2_ every 5 min. The sample was then centrifuged (100×g, 2 min, 4°C), and the resulting pellet of tubules resuspended in fresh HEPES-buffered physiological saline and stirred on ice for 30 min. After stirring, the sample was filtered through a 250 µm nylon mesh, and the filtered tubules were collected and rinsed at 4°C. This preparation resulted in a 95% pure suspension of thick ascending limbs.

### NO bioavailability measurement

Intracellular NO bioavailability in thick ascending limb suspensions was measured using the dye DAF-FM. A 5 mmol/l stock solution was prepared daily in dimethyl sulfoxide (DMSO). Tubules were loaded by adding 5 µmol/l DAF-FM to the medium while being stirred on ice during suspension preparation [33]. Two different methods were used to assay NO bioavailability. In the first, the effects of resveratrol and vehicle on NO bioavailability were measured in separate aliquots of a single suspension. After loading the tubules with dye, the suspension was centrifuged (100×g, 2 min, 37°C) and the tubules were resuspended in 2 mM Ca^2+^ media with 100 µmol/l L-arginine and gassed with 100% O_2_ at 37°C. They were then incubated and rinsed repeatedly every 5 min at 37°C for 15 min to cleave the dye and wash out the acetylated form. After the last rinse, the suspension was split into two 1500-µl aliquots, loaded into different cuvettes, and incubated for 5 min at 37°C before acquiring measurements. Then, vehicle or resveratrol was added and the increase in fluorescence over time was measured for 20 sec, at 1-min intervals, over 5 min. Dye was excited at 485 nm and fluorescence collected at 515 nm. After these measurements, the suspensions were transferred to 2-ml tubes, cooled on ice, and the tubules were recovered by centrifugation for protein measurements. Data from both vehicle and resveratrol treatments were analyzed by linear regression and expressed as resveratrol-induced arbitrary fluorescence units (AFU) per mg protein per min. The DMSO concentration never exceeded 0.1%. In these experiments, when the effects of different drugs were assessed, the drugs were added just after aliquots were separated and kept in the media until the end of the experiment.

In the second method, resveratrol-induced NO production was continuously measured over time on a single aliquot. For this purpose, thick ascending limb suspensions were loaded with DAF-FM and washed as described above. Then the whole suspension was transferred to a cuvette and stirred in the spectrofluorometer for 5 min with the shutter closed for stabilization, followed by a 2-min equilibration period with the shutter open. Basal fluorescence was collected for 4 additional min, resveratrol was added, and the fluorescence was collected again for 4 min. In these experiments, drugs other than resveratrol were added during the 5-min stabilization period.

**Figure 1:**
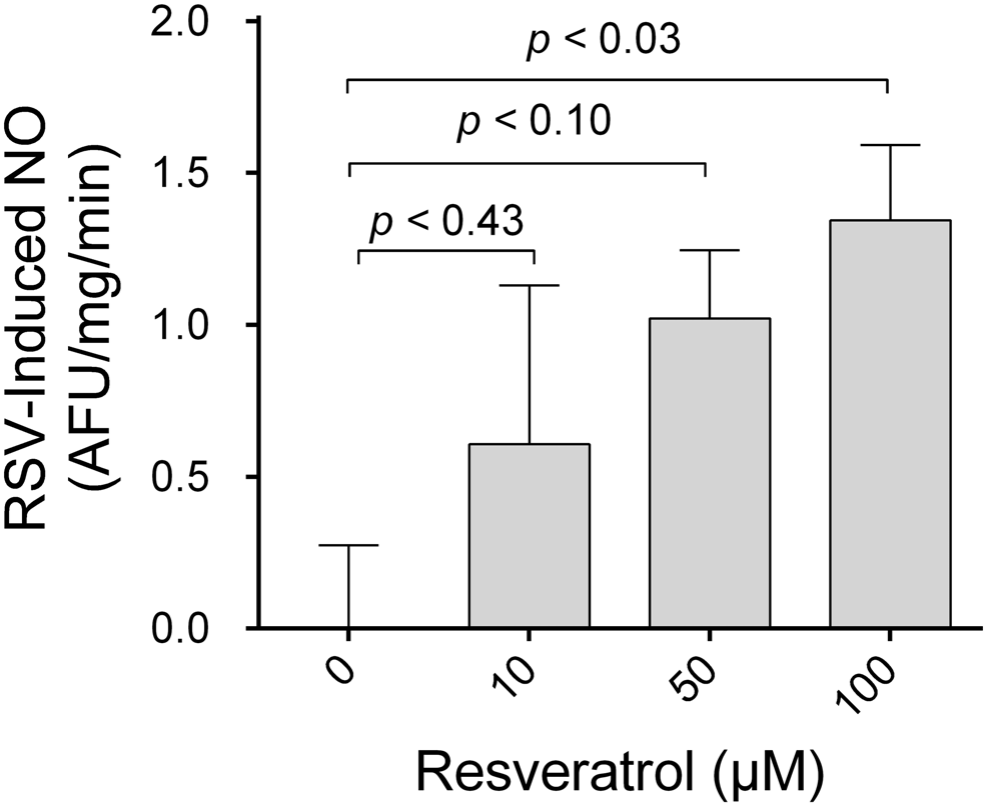
Concentration dependence of resveratrol’s effect on NO bioavailability. Adjusted *p-*values for multiple comparisons using Dunnett’s posttest are reported.

**Figure 2:**
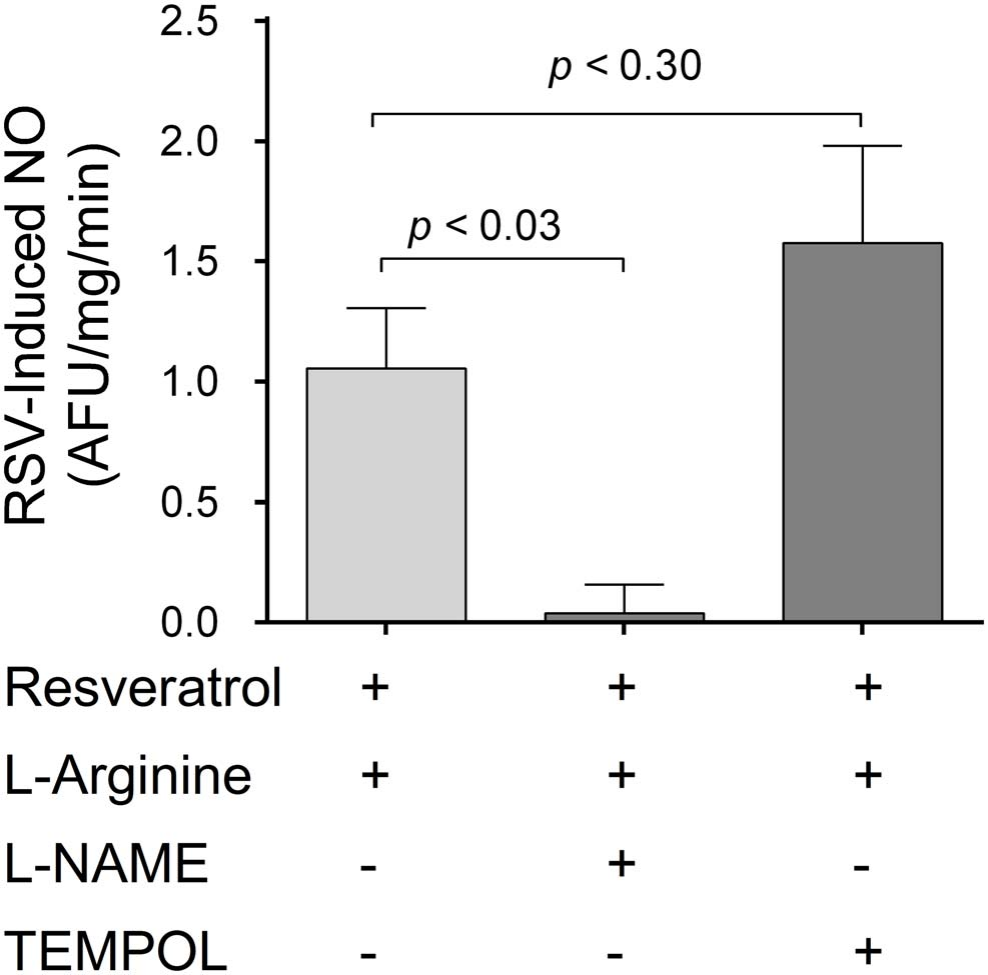
Effect of L-NAME and tempol on resveratrol-stimulated NO. Adjusted *p-*values for multiple comparisons using Dunnett’s posttest are reported.

Data depicted in Figures 1 and 2 were obtained with a spectrofluorometer Perkin Elmer 650-40, and the analogic signal recorded using a Power lab chart recorder with Labchart 7 software, while those for all other figures were obtained with a Hitachi F-2700 spectrofluorometer and the digital signal recorded and analyzed using FL Solution 4.1 software.

In the experiments in which the effect of removing extracellular Ca^2+^ on resveratrol-induced NO were measured, 2.0 mM Ca^2+^ media was substituted with Ca^2+^-free media. This medium was supplemented with either 100 µmol/l or 200 µmol/l EGTA. Results with either 100 or 200 µmol/l EGTA were not different so the data were pooled together.

### Thick ascending limb isolation and microperfusion

Single medullary thick ascending limbs were isolated and perfused as previously described [34], [35]. Briefly, animals were anesthetized, and the abdominal cavity opened. The left kidney was bathed in an ice-cold 150 mmol/l NaCl solution, immediately removed and placed in HEPES-buffered physiological saline at 4°C. Coronal slices were cut and individual thick ascending limbs isolated from the outer medulla under a stereomicroscope at 4°C. Tubule length ranged from 0.8 to 1.0 mm. A single thick ascending limb was transferred to a temperature-regulated chamber and perfused using concentric glass pipettes at 37±1°C. The bath was exchanged at 1 ml/min.

### Ca_i_ measurements

Individually perfused thick ascending limbs were loaded for 30 min with 1 µmol/l FURA 2-AM dissolved in 2 mM Ca media, followed by a 30-min washing/cleavage period using dye-free 2 mM Ca^2+^ media. To study the effects of resveratrol in the absence of extracellular calcium, the 30-min washing/cleavage period was performed using Ca^2+^-free media with 200 µmol/l EGTA in both luminal and basolateral sides.

For measurements, the dye was alternatively excited at 340 nm and 380 nm and fluorescence collected at 510 nm. Thick ascending limbs were digitally imaged using an image intensifier adapted to a charge-coupled device camera, and data were recorded using MetaFluor 7 Fluorescence Ratio Imaging Software (Universal Imaging, Downingtown, PA). Resting Ca_i_ levels were measured for 5 min taking images every 30 sec. Then, 100 µmol/l resveratrol was added to the basolateral bath dissolved in either Ca^2+^-containing or Ca^2+^-free media, and Ca_i_ was measured again. At the end of each individual experiment, a 2-point calibration was performed using Ca^2+^-free media with 5 mmol/l EGTA and 2 mmol/l Ca^2+^ media added with 10 µmol/l 4-Br-A23187. Ca_i_ was calculated using the following equation as previously described:
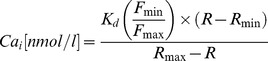
Where K_d_ is the dissociation constant of FURA 2 for Ca^2+^ (224 nmol/l); R is the fluorescence ratio following 340-nm and 380-nm excitation respectively; F_min_ is the minimum fluorescence intensity at 380-nm excitation during EGTA treatment; F_max_ is the maximum fluorescence intensity at 380-nm excitation during 4-Br-A23187 treatment; R_min_ is the minimum fluorescence ratio following 340-nm and 380-nm excitation respectively during EGTA and R_max_ is the maximum fluorescence ratio following 340-nm and 380-nm excitation respectively during 4-Br-A23187 treatment.

### Western blot for SIRT1

Tubules were dissolved in lysis buffer and 25, 50 and 100 µg protein were loaded onto a 6% polyacrylamide gel. Electrophoresis was performed and membranes were incubated with a primary mouse monoclonal anti-SIRT1 antibody (Cell Signaling Technology, Danvers, MA) (diluted 1∶1000) for 2 hr; washed, and reincubated for 1 hr with a horseradish peroxidase (HRP)-conjugated anti-rabbit IgG (diluted 1∶1000) (Amersham Pharmacia Biotech). The membranes were incubated with a luminol-based chemiluminescent HRP substrate (Pierce Biotechnology, Rockford, IL) and exposed to a radiographic film for 5 min. Films were scanned and densitometry of the blots was performed using a customized program. As a loading control, tubulin was detected using rabbit polyclonal anti-β tubulin antibody (Abcam, Cambridge, Ma) (diluted 1∶10000) and a horseradish peroxidase (HRP)-conjugated anti-rabbit IgG (diluted 1∶5000) (Amersham Pharmacia Biotech).

### SIRT1 activity measurement

The SIRT1 Direct Fluorescent Screening Assay Kit (Cayman Chemical Company, Ann Arbor, MI) was used as follows: tubules were dissolved in lysis buffer supplemented with 100 µM trichostatin A (a Zn^2+^-dependent deacetylases inhibitor) and kept on ice. Protein concentration was measured and samples were diluted to 2 µg/µl. SIRT1 activity was assessed using 20 µg protein according to the manufacturer recommendations. Basal and resveratrol-induced SITR1 activity was assayed by adding either vehicle or 100 µmol/l resveratrol. Lysates supplemented with human-recombinant SIRT1 (hrSIRT1) were run as positive controls.

### Statistical analysis

All data were analyzed using GraphPad Prism, version 6.02. Results are expressed as the arithmetic mean ± the standard error of the mean. For multiple comparisons, 1-way ANOVA followed by Dunnett’s post-test analyses were performed. Adjusted *p values* for all groups compared to controls are reported in the text. For comparison of 2 means, Student T-tests were used. The *p values* were calculated using 2-tailed tests in all cases, and paired or unpaired test were used where appropriate. *p<*0.05 was considered significant.

## Results

First we measured the effect of different resveratrol concentrations (10, 50 and 100 µmol/l) or vehicle (0.1% DMSO) on DAF-FM fluorescence in thick ascending limb suspensions. We found that 100 µmol/l resveratrol increased DAF-FM signal by 1.3±0.2 AFU/mg/min as compared to vehicle (p<0.03; Figure 1) while lower concentrations had no significant effect. Thus, we used 100 µmol/l resveratrol in all other experiments.

An increase in DAF-FM fluorescence, a surrogate of NO availability, can be either due to enhanced NO production or decreased NO degradation. Superoxide anion (O_2_^−^) scavenges NO, thus reductions in O_2_^−^ by resveratrol could augment DAF-FM fluorescence. To test this, we measured resveratrol-induced DAF-FM fluorescence in the presence of either NOS inhibitor L-NAME or the O_2_^−^ scavenger tempol (100 µmol/l). We found that L-NAME blunted resveratrol-stimulated NO by 96±11% (*p<*0.03; Figure 2). In contrast, scavenging O_2_^−^ with tempol neither increased nor decreased the ability of resveratrol to enhance NO bioavailability (Figure 2). L-NAME and tempol data taken together indicate that resveratrol stimulates NOS activity in the thick ascending limb.

**Figure 3:**
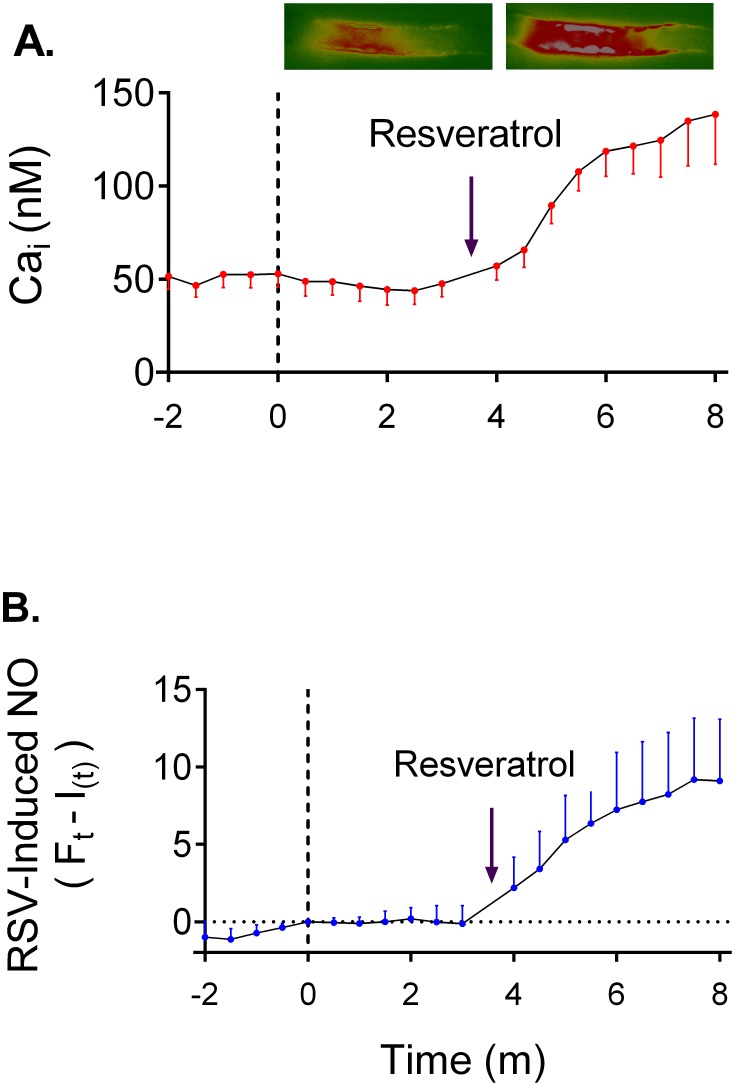
Timeframe of resveratrol-induced increased in Ca_i_ and NO. **A)** Effect of resveratrol on Ca_i_ in individually perfused tubules. Pictures show the FURA 2 ratiometric signal before and after addition of resveratrol. Resveratrol-induced Ca_i_ was measure by comparing the average after 4 minutes of addition of resveratrol to the average at time 0. Values and statistics reported in the text. **B)** Effect of resveratrol on NO production in tick ascending limb suspensions. For graphing purposes, NO production values were averaged every 30 sec and the resveratrol-induced NO defined: 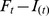. 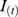 is the resveratrol-independent fluorescent signal defined: 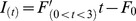; where 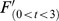 is the first derivative of 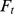 evaluated from time 0 to 3, and 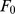 is fluorescence at time 0. No statistical analysis was conducted on this data set (n = 7).

We next investigated the mechanism by which resveratrol increases NO production. In several cell types, resveratrol increases Ca_i_ [31], [36]–[38], and NOS3 is activated by elevations in Ca_i_ [7], [8]. Thus, we studied whether resveratrol raises Ca_i_ in isolated perfused thick ascending limbs. We found that basolateral addition of 100 µmol/l resveratrol produced a sustained elevation in Ca_i_ from 48±7 to 135±24 nmol/l after 4 min (p<0.01, n = 5; Figure 3-A). This increase in Ca_i_ parallels the increase in NO production measured separately in thick ascending limb suspensions (Figure 3-B).

**Figure 4:**
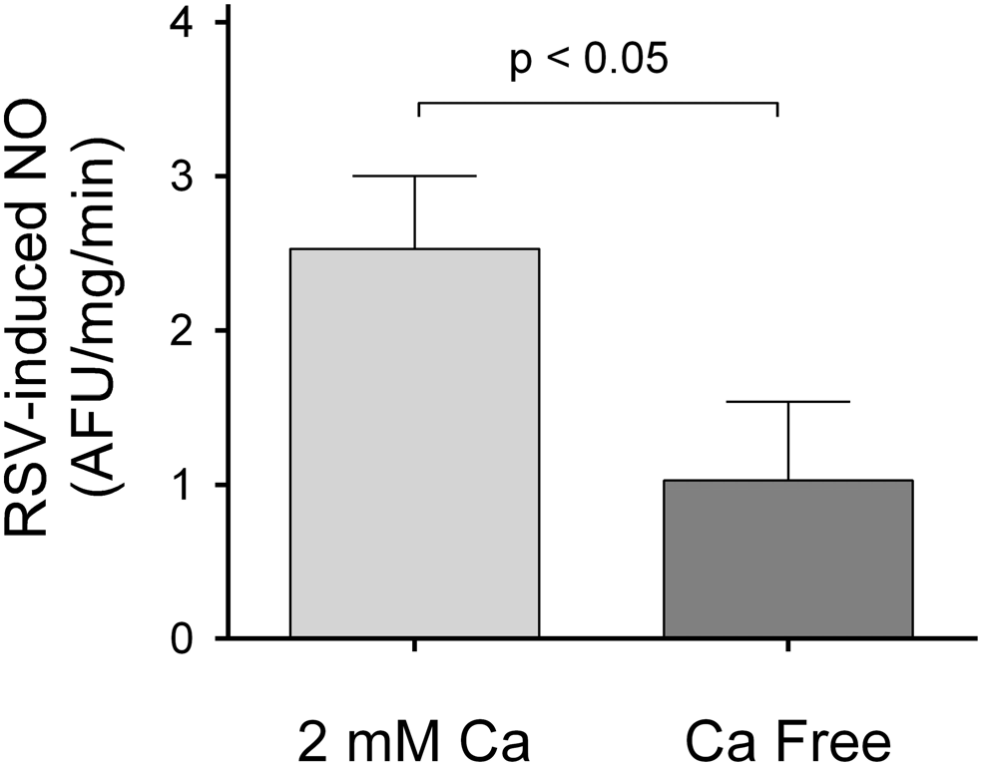
Effect of a Ca^2+^-free media on the NO response to resveratrol. (n = 9 for controls and 11 for Ca^2+^-free).

To examine whether the resveratrol-induced increase in Ca_i_ was due to release from intracellular stores or influx of extracellular Ca^2+^, we measured resveratrol-induced NO production in Ca^2+^-free media. Under these conditions, the resveratrol-induced NO was blunted by 60% (p<0.05), from 2.5±0.5 AFU/mg/min (n = 9) to 1.0±0.5 AFU/mg/min (n = 11) (Figure 4). In separate experiment, we measured the effects of resveratrol in Ca_i_ in isolated perfused tubules bathed on Ca^2+^-free solution. We found that in the absence of extracellular Ca^2+^, resveratrol increased Ca_i_ only by 17±4 nmol/l after 4 min (p<0.01, n = 5), a response 80% lower than in a Ca^2+^-containing media. These results indicate that the NO-response to resveratrol largely depends on Ca^2+^ influx.

**Figure 5:**
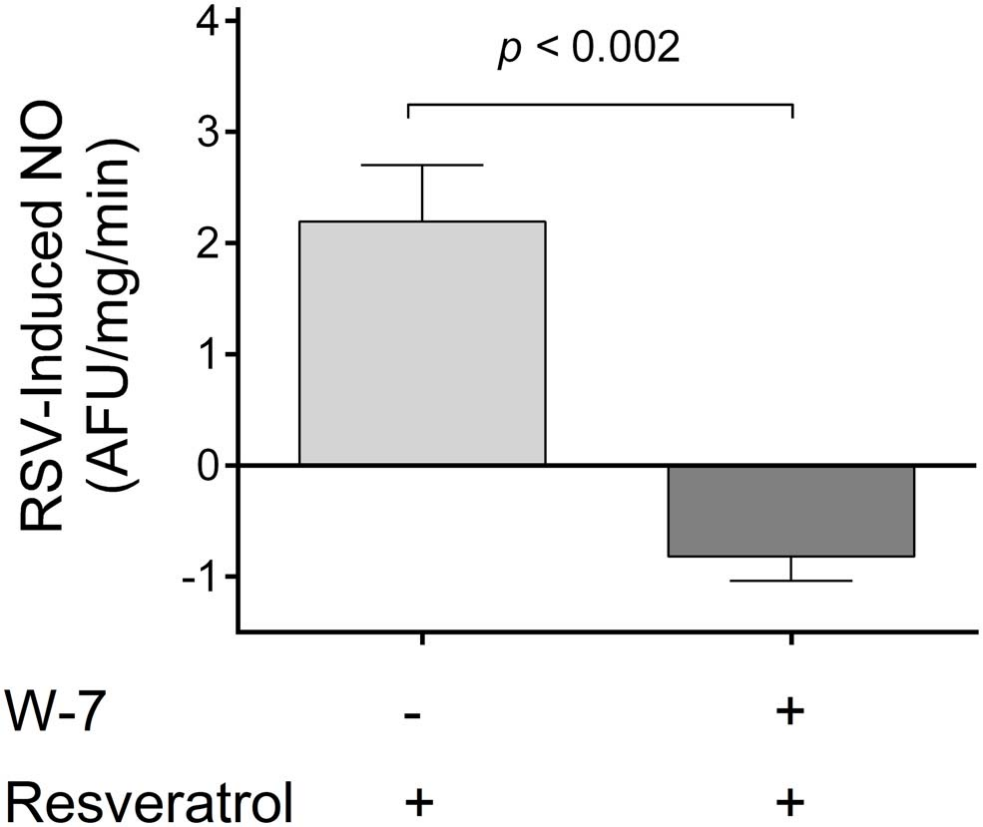
Effect of the calmodulin inhibitor W-7 on the NO response to resveratrol. (n = 5 for controls and 4 for W-7).

The most likely mechanism by which elevations in Ca_i_ increase NO production is *via* calmodulin. Thus, we tested whether calmodulin participates in the activation of NOS by resveratrol in the thick ascending limb. To do this, we measured resveratrol-induced NO production in the presence of 100 µmol/l calmodulin inhibitor W-7. We found that inhibition of calmodulin completely blocked the NO response to resveratrol; −0.8±0.2 AFU/mg/min (n = 4) vs. 2.2±0.5 AFU/mg/min in the controls (n = 5; *p<*0.002; Figure 5). Taken together, these data indicate that resveratrol enhances NO production *via* Ca^2+^/calmodulin upon Ca^2+^ influx from the extracellular medium.

In addition to Ca_i_, some enzymes that can mediate increases in NO such as SIRT1 and AMPK, have been reported to be stimulated by resveratrol [9], [39], [40]. Thus, we explored SIRT1 and AMPK pathways in the thick ascending limb.

**Figure 6:**
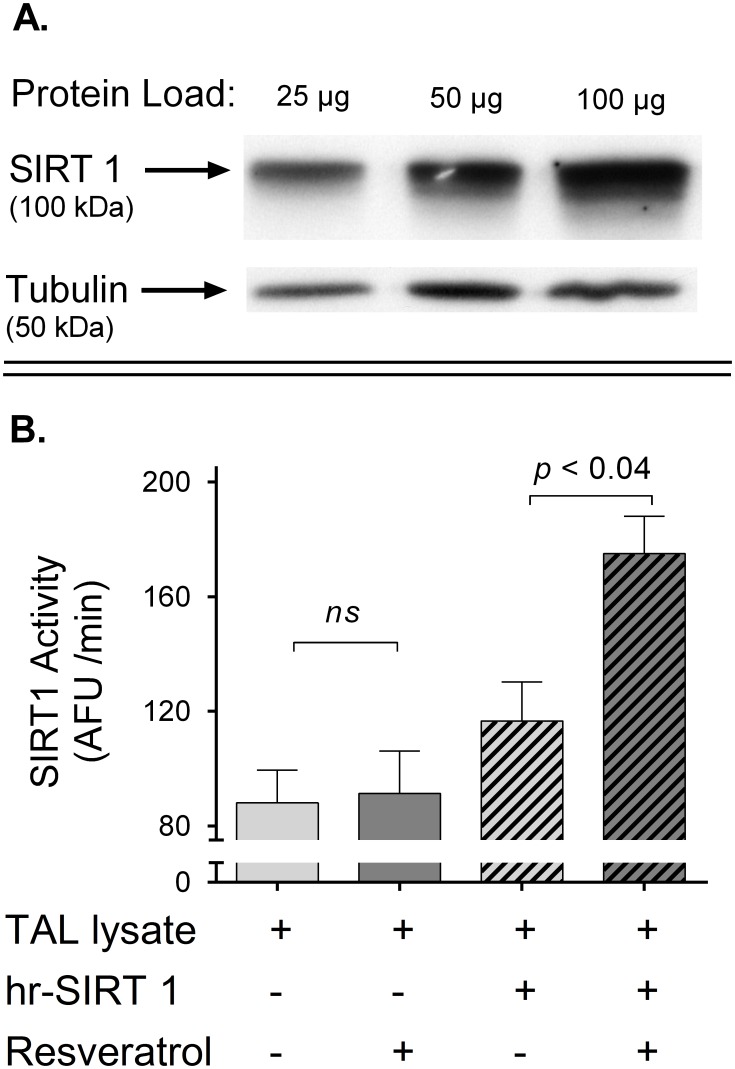
Effects of resveratrol on endogenous SIRT1. **A)** SIRT1 expression in thick ascending limb (TAL) lysates obtained from TAL suspensions. **B)** Effect of resveratrol on SIRT1 activity in TAL lysates. Lysates supplemented with human-recombinant SIRT1 (hrSIRT1) were run as positive controls.

We first measured SIRT1 expression by Western blot in thick ascending limb homogenates using a monoclonal antibody. We found a single band at 100 kDa, indicating that thick ascending limbs express SIRT1 (Figure 6-A). Next we measured whether resveratrol stimulates SIRT1 activity. We found that the addition of resveratrol to thick ascending limb lysates did not increase histone deacetylase activity (91±15 vs. 88±11 AFU/min in the vehicle; ns, n = 4). To test for the presence of endogenous inhibitors or non-specific effects of trichostatin A, lysates in which we added human recombinant SIRT1 served as positive controls. Resveratrol enhanced human recombinant SIRT1 activity from 117±14 to 175±13 AFU/min (p<0.05; Figure 6-B) in the positive controls, indicating that we were able to measure SIRT1 activity in our system. These data show that resveratrol does not stimulate SIRT1 activity acutely in the thick ascending limb, thus it is unlikely that the NO-response to resveratrol depends on this pathway.

**Figure 7:**
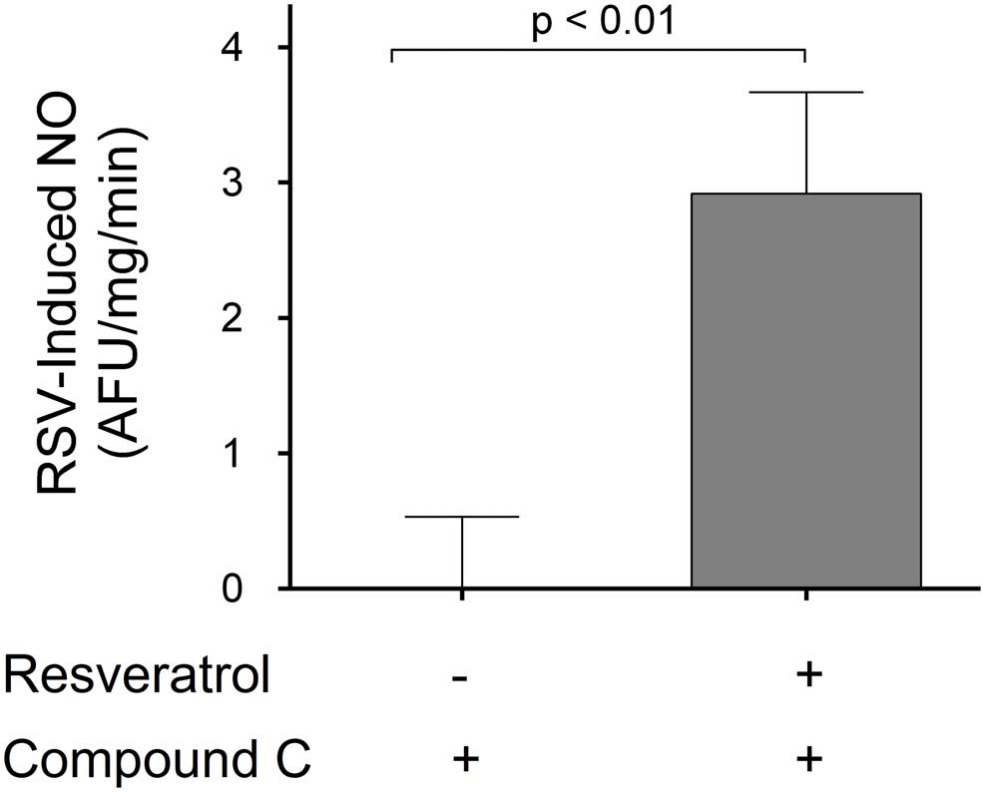
Effect of Compound C (AMPK inhibitor) on the NO response to resveratrol (n = 6, compared to vehicle).

To study the involvement of AMPK in the resveratrol-induced NO production in the thick ascending limb, we pre-incubated suspensions with the AMPK inhibitor compound C (40 µmol/l). Under these conditions, addition of resveratrol increased NO production by 2.9±0.8 AFU/mg/min compared to vehicle (p<0.01; n = 6; Figure 7). These results indicate that AMPK is not a downstream effector of resveratrol in this tissue.

## Discussion

We hypothesized that resveratrol stimulates NO production by thick ascending limbs *via* a Ca^2+^/calmodulin-dependent mechanism.

To test our hypothesis we first measured whether resveratrol acutely increased NO bioavailability in thick ascending limb suspensions. We evaluated the effects of vehicle (0.1% DMSO) or 10, 50 and 100 µmol/l resveratrol on DAF-FM fluorescence. In these experiments, only 100 µmol/l resveratrol significantly increased NO bioavailability as compared to vehicle. Similar to our results, the cGMP levels in vasculature derived cells were reported to be elevated after a 2-min treatment with 100 µmol/l resveratrol, while lower doses had no significant effect [41].

Our results regarding resveratrol-induced NO in renal tissue are consistent with a previous report, in which intravenous infusion of resveratrol increased renal NO in rats [21]. In addition, systemic treatment with resveratrol increased urinary cGMP [20] and renal blood flow [42] and decreased renal vascular resistance[42]. All these effects were mediated by NO since co-administration of the NOS inhibitor L-NAME blunted them.

An increase in NO bioavailability can be either due to enhanced production or decreased degradation. Superoxide anion (O_2_^−^) scavenges NO, thus reductions in O_2_^−^ levels would augment NO bioavailability. The renal medulla is the primary source of O_2_^−^ in the kidney. In addition NADPH oxidase is the main source of O_2_^−^ in the thick ascending limb [43], and previous reports show that resveratrol inhibits its activity in the vasculature [26].

To test whether the increase in DAF-FM signal was due to elevated NO production, decreased O_2_^−^ levels, or both; we measured resveratrol-induced NO in the presence of either L-NAME or the O_2_^−^ scavenger tempol. L-NAME blunted resveratrol-stimulated NO by 96%. In contrast, scavenging O_2_^−^ with tempol has not a significant effect on the ability of resveratrol to enhance NO bioavailability.

These data suggest that the increase in NO levels elicited by resveratrol in thick ascending limbs is due to stimulation of NOS activity rather than scavenging O_2_^−^ or inhibiting NADPH oxidase. If resveratrol would have increased NO simply by reducing O_2_^−^, one would have expected the effect of resveratrol to be blunted in the presence of tempol. Our results show just the opposite, a trend to a higher response in the absence of O_2_^−^. Thus our data suggest that resveratrol does not increase NO bioavailability by scavenging O_2_^−^. Furthermore, since there was a trend for tempol to enhance the effect of resveratrol on NO bioavailability, our data suggest that endogenous O_2_^−^ scavenges part of the resveratrol-induced NO. Our results are similar to those of a previous report in which systemic infusion of resveratrol produced an endothelium-dependent dilation of the renal vasculature mediated mainly by NO [42]. Reductions in reactive oxygen species had a small but significant effect [42]. Similarly, other reports in the vasculature support the idea that the ability of resveratrol to induce vasodilation largely depend on its capacity of stimulate NO production [44]–[46].

Since our data show that resveratrol stimulates NOS activity, we next investigated the mechanism involved. Originally it was proposed that resveratrol might enhance NO production by binding directly to NOS; however, it had no effect on NO production by purified NOS3 in solution or in rat aortic homogenates [26], [31]. Such data indicate that resveratrol enhances NO production *via* signaling cascades. In several cell types, resveratrol increases Ca_i_ [31], [36]–[38], and NOS are activated by elevations in Ca_i_ [7]. Thus, we studied whether resveratrol raises Ca_i_ in isolated perfused thick ascending limbs. We found that basolateral addition of resveratrol produced a sustained elevation in Ca_i_. This increase in Ca_i_ parallels the increase in NO production measured separately in thick ascending limb suspensions.

Then, to examine whether the resveratrol-induced increase in Ca_i_ was due to release from intracellular stores or influx of extracellular Ca^2+^, we measured resveratrol-induced increases in Ca_i_ and NO production in a Ca^2+^-free media. Under these conditions, the increases in Ca_i_ and NO production were blunted by 80% and 60% respectively, suggesting that the NO-response to resveratrol largely depends on Ca^2+^ influx.

The most likely mechanism by which Ca_i_ increases NO production is *via* calmodulin. The Ca^2+^/calmodulin complex binds to NOS, facilitating the electron flux throughout its domains and subsequently increasing NO production [7]. Thus, we tested whether calmodulin participates in the activation of NOS by resveratrol in the thick ascending limb. To do this, we measured resveratrol-induced NO production in the presence W-7, a calmodulin inhibitor. We found that inhibition of calmodulin completely blocked the NO response to resveratrol. Taken together, these data indicate that resveratrol enhances NO production *via* Ca^2+^/calmodulin upon Ca^2+^ influx from the extracellular medium.

Our results concerning Ca^2+^ are consistent with previous reports in epithelial [31] and chromaffin cells [37] in which resveratrol increased NO production in a Ca^2+^-dependent fashion; however, none of those reports evaluated the effects of calmodulin inhibition.

Resveratrol-dependent increases in Ca_i_ have been reported in several cell-lines including human prostate cancer cells [38], vascular smooth muscle cells [36], [47], epithelial cells [31] and chromafin cells [37]. However, the mechanisms by which these elevations in Ca_i_ occur remain elusive. For instance, in vascular cells [36], [47] the resveratrol-induced elevation in Ca_i_ seems to be due to activation of several types of transmembrane Ca^2+^-permeable channels, while in a cancer cell-line, Ca^2+^ apparently is released from the endoplasmic reticulum [38]. On the other hand, endothelial cells exhibit two different positive responses to resveratrol [31]. In about 30% of the assayed cells, resveratrol produced a transient increase in Ca_i_ due to release from intracellular stores followed by a sustained increase due to Ca^2+^ influx from the extracellular space. In 50% of the cells, resveratrol produced a slow and sustained rise in Ca_i_ similar to that observed by us in isolated perfused thick ascending limbs. The explanation for these apparently disparate data remains elusive; however, it is possible that resveratrol acts to elevate Ca_i_ through different simultaneous mechanisms.

In addition to Ca_i_, some enzymes that can mediate increases in NO such as SIRT1 and AMPK, have been reported to be stimulated by resveratrol [9], [39], [40]. Thus, we explored SIRT1 and AMPK pathways in the thick ascending limb. Given that there were no previous reports of SIRT1 presence in the thick ascending limb, we first analyzed SIRT1 expression by Western blots. We found that thick ascending limbs express SIRT1 (Figure 6-A). We next measured whether resveratrol stimulates SIRT1 activity in thick ascending limbs lysates. We found that the addition of resveratrol did not increase endogenous SIRT1 (Figure 6-B). In these experiments, lysates added with human recombinant SIRT1 served as positive controls (Figure 6-B). Given that these data show that resveratrol does not stimulate endogenous SIRT1 activity acutely, it is unlikely that the NO-response to resveratrol depends on this pathway. This is consistent with a previous study in epithelial cells, in which SIRT1 inhibition had no effect on resveratrol-induced NO [31].

Finally, to study the involvement of AMPK in the resveratrol-induced NO production in the thick ascending limb, we measured resveratrol-induced NO in the presence of AMPK inhibitor compound C. We found that AMPK inhibition does not blunt the acute response to resveratrol (Figure 7). These results indicate that AMPK is not a downstream effector of resveratrol in this tissue acutely.

As opposed to the thick ascending limb, on human umbilical vein endothelial cells, AMPK inhibition exerts a mild inhibitory effect in acute resveratrol-induced NO production independently of Ca_i_ levels [31]. This is likely due to the activation of AMPK by phosphorylation at T172 [28]. However, when other kinases such as Ca^2+^/calmodulin-dependent protein kinase are activated, AMPK is unlikely to play a major role in NOS3 regulation [8].

AMPK and SIRT1 could be important though in the long-term effects of resveratrol which are mediated by activation of several different genes [29], [48], [49]. In fact AMPK and SIRT1 have been reported to have cooperative effects to activate NOS3 [50], [51], as for instance, resveratrol supplementation of db/db mice, prevents renal damage by increasing AMPK phosphorylation and SIRT1 signaling [24].

The renal medulla is the main site for NO production in the kidney, where NOS activity is fine-tuned by several mechanisms acting simultaneously [52], [53]. In the thick ascending limb NO reduces NaCl reabsorption. Defective NO production and/or signaling in the renal medulla, contribute to many forms of hypertension including angiotensin II-dependent hypertension and the increase in blood pressure in spontaneously hypertensive rats [1]–[3]. Early supplementation with resveratrol prevents the increase in blood pressure in these models [45], [54], [55]. However, resveratrol does not reduce blood pressure once hypertension is stablished [44], [56]–[59]. This may be due to morphological changes that contribute to sustain the elevated blood pressure. Such data indicate that resveratrol may be used as a preventive drug. In fact, resveratrol supplementation prevents kidney damage in several experimental models [20], [21], [60]–[65].

Our current work provides a mechanistic explanation of how resveratrol acutely increases NO production in renal tissue obtained from healthy animals. Future studies should better characterize the pharmacological properties of resveratrol and evaluate the feasibility of resveratrol supplementation.

In summary, our experiments show that resveratrol acutely increases NO production in the thick ascending limbs of healthy rats. This increase depends on the activation of NOS by calmodulin upon Ca^2+^ influxes from the extracellular environment. AMPK and SIRT1 are not involved.
